# Supplementary material for: Untargeted Metabolomic Analysis of Lactation-Stage-Matched Human and Bovine Milk Samples at 2 Weeks Postnatal
Source: Nutrients. 2023 Aug 29;15(17):3768. doi: 10.3390/nu15173768 (PMC10490210; doi:10.3390/nu15173768)
Supplement: Supplementary file 1 [file nutrients-15-03768-s001.zip › nutrients-2553025-supplementary.pdf]

Supplementary Material—Figures

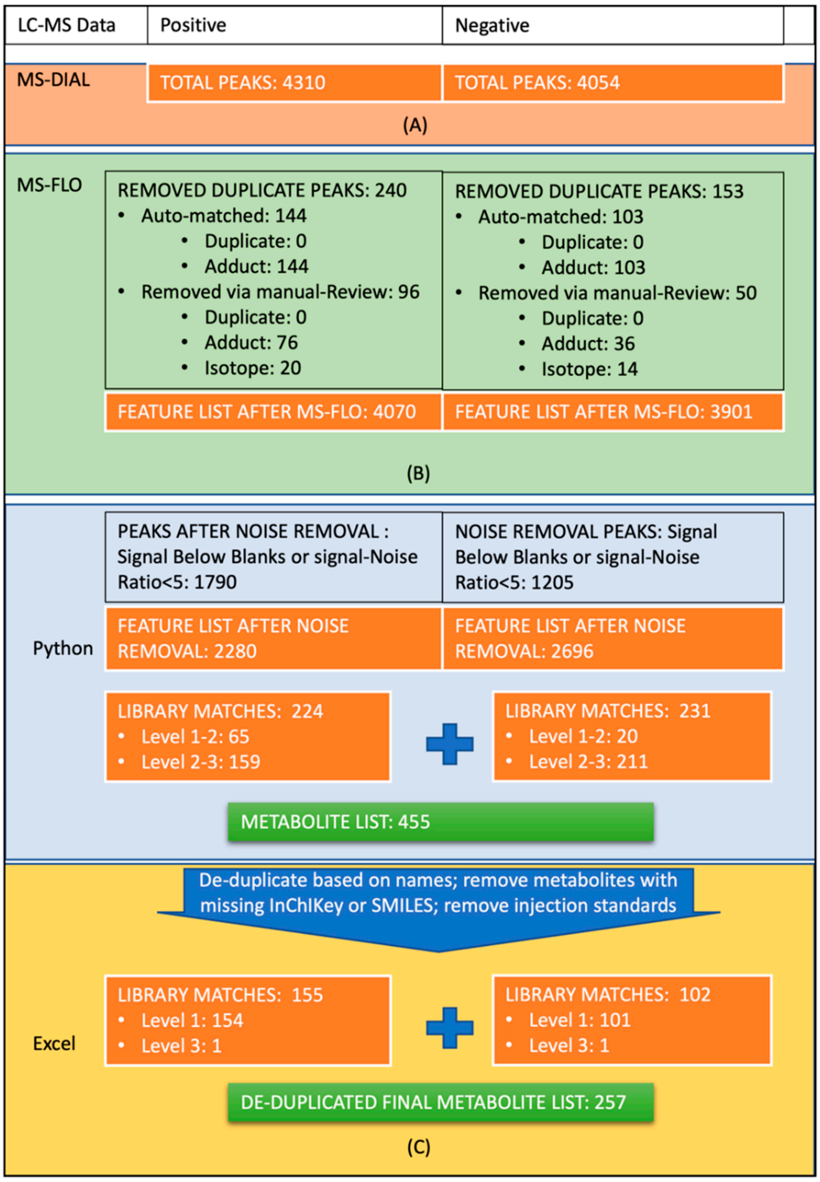

Figure S1. Consort diagram for data processing and metabolites identification. A consort diagram, also known as a flow diagram, is a graphical representation used in medical and clinical research to depict the flow of data. (A) Orange part represents data processing with MS-DIAL, 4,310 features were identified initially via positive mode while 4,054 were identified in negative mode. (B) Green part illustrates results got from MS-FLO, which optimized MS-DIAL results by reducing potential duplicates, adducts, and isotopes. 4,070 positive features and 3,901 features left separately after MS-FLO. (C) Python (blue section) was then used for noise removal and metabolites identification, the rest metabolites which are duplicated or biological unlikely were manually removed (orange section). As a result, 155 identified unique positive features and 102 unique identified negative features (257 in total) were included in the final table. 154 features (positive) and 101 features (negative) were identified with a confidence level 1, while the numbers are 1 and 1 for level 3 identification.

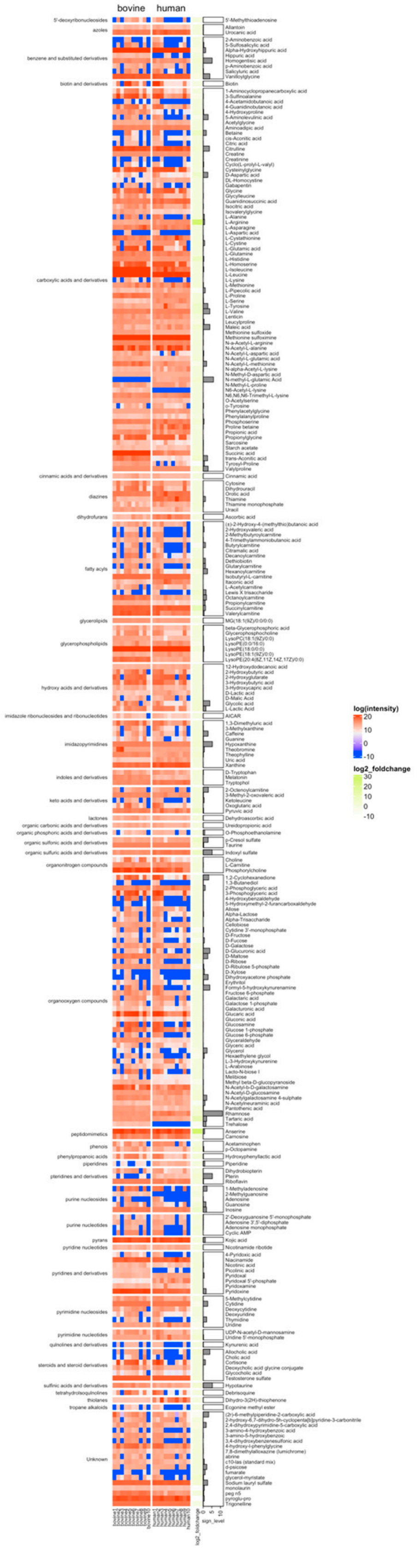

Figure S2. Heatmap for all 257 identified unique metabolites, whose columns are split by sample type (human and bovine) and rows are split by metabolite classes. Rows are separated by classes in

HMDB and columns are separated by sample type. The main heatmap scale ranges from -10 to 20 on a log2 scale. Red means high intensity while blue indicates low intensity. Log2 fold change is represented by the single-column heat bar on the right side of the map, in which yellow means big fold change and while means the opposite. The bar plot on the rightest section represents a significant level (i.e., -log10(FDR)).

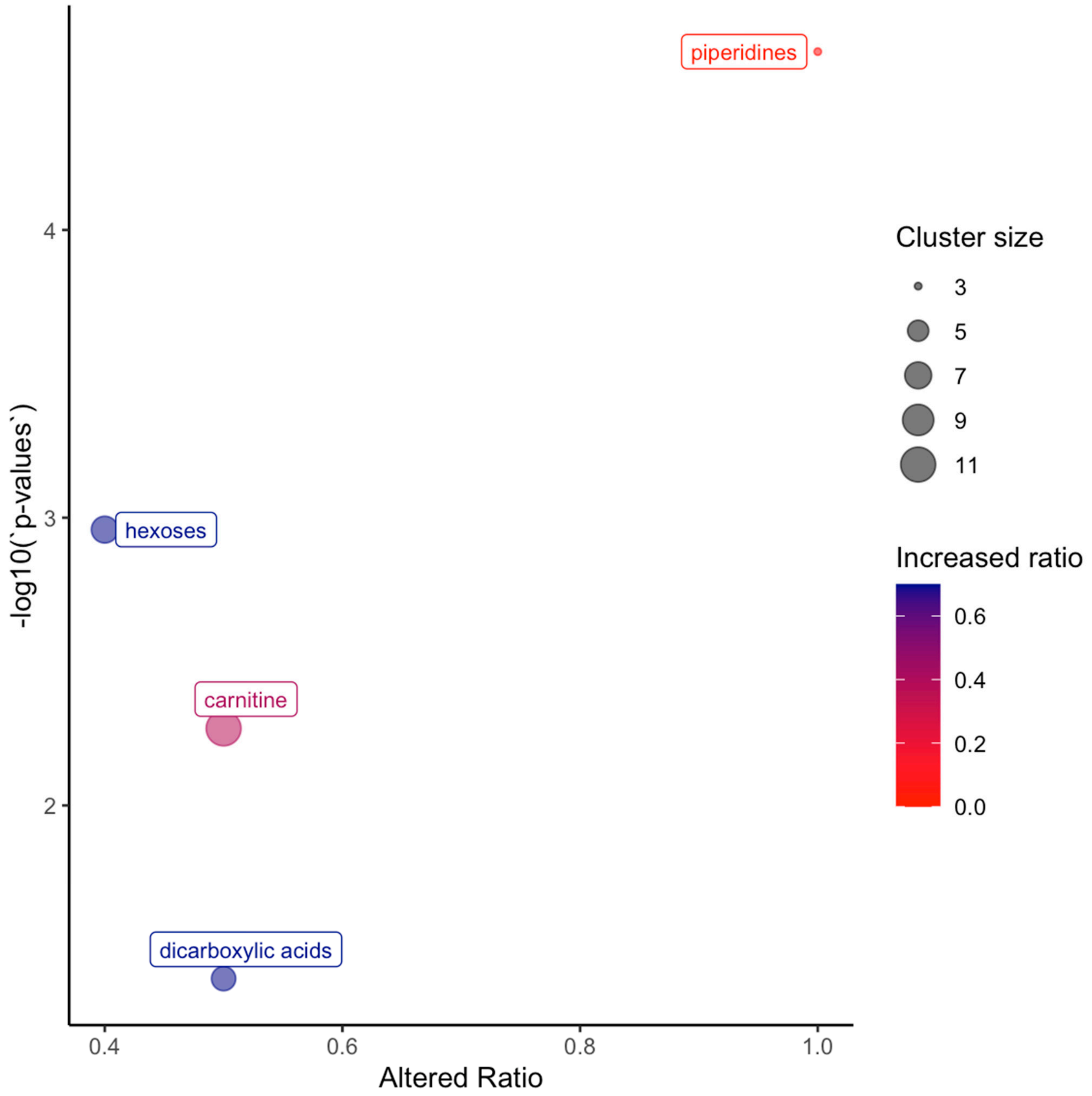

Figure S3. Chemical enrichment analysis results. This is the enrichment statistics for human milk vs. bovine milk. Each cluster represents altered chemical class of metabolites ($p<0.05$). Cluster size stands for total number of metabolites. Cluster color represents the proportion of increased or decreased metabolites. Blue means higher in human milk, red means higher in bovine milk. Color in between refer to mixed population of metabolites manifesting both higher and lower levels in human/bovine milk. Y-axis indicates the most significantly altered clusters on the top. X-axis indicates the percentage of metabolites in a cluster associated with a raw p-value < 0.05. Kolmogorov-Smirnov test was employed for the statistics. Only significantly different enrichment clusters (raw $p< 0.05$) were shown. The node sizes stand for the total number of metabolites in each cluster set.

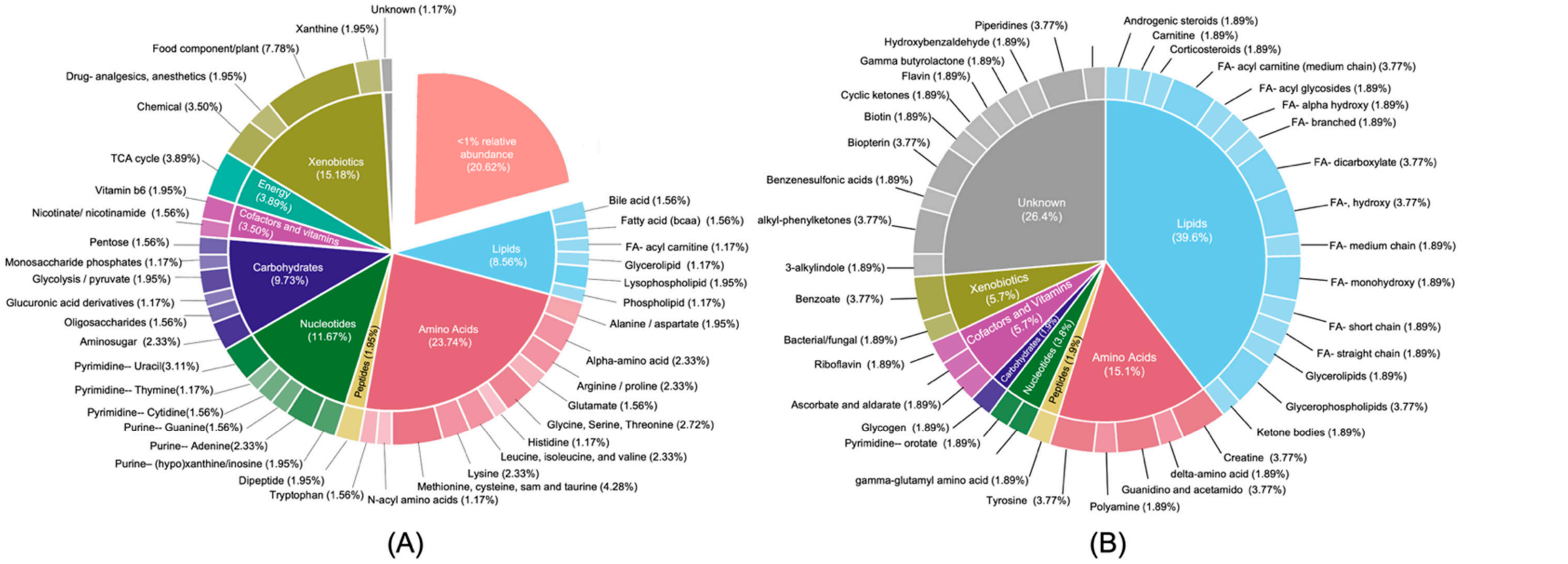

Figure S4. Detailed Pie Chart of overall composition. (**A**) **Pie Chart of Complete Composition.** Indicates the 10 classes of metabolites discovered in both human and bovine milk samples with labels. (**B**) **Pie Chart of Less than 1% Abundance.** Indicates metabolites located within 8 classes with detailed labels. Color intensity of subclasses indicate a direct relationship with abundance of metabolite found.

Supplementary Material—Tables

Supplementary Table S1. Classification of identified metabolites.

Class	%	Count	Contents	%
Amino Acids	23.7%	11	Methionine, Cysteine, Sam and Taurine	4.3%
			Glycine, Serine, and Threonine	2.7%
			Arginine and Proline	2.3%
			Lysine	2.3%
			Leucine, Isoleucine, and Valine	2.3%
			Alpha- amino acids	2.3%
			Alanine and Aspartate	2.0%
			Glutamate	1.6%
			Tryptophan	1.6%
			N-acyl amino acids	1.2%
			Histidine	1.2%
<1% relative abundance	20.6%	1	<1% relative abundance	20.6%
Xenobiotics	15.2%	4	Food component/ plant	7.8%
			Chemical	3.5%
			Xanthine metabolism	2.0%
			Drugs- analgesics, anesthetics	2.0%
Nucleotides	11.7%	6	Pyrimidine metabolism, uracil	3.1%
			Purine metabolism, adenine	2.3%
			Purine metabolism, (hypo)xanthine/inosine	2.0%
			Purine metabolism, guanine	1.6%
			Pyrimidine metabolism, cytidine	1.6%
			Pyrimidine metabolism, thymine	1.2%
Carbohydrates	9.7%	6	Aminosugar	2.3%
			Glycolysis, gluconeogenesis, and pyruvate	2.0%
			Disaccharides and Oligosaccharides	1.6%
			Pentose metabolism	1.6%
			Glucoronic acid derivatives	1.2%
			Monosaccharide phosphates	1.2%
Lipids	8.6%	6	Lysophospholipid	2.0%
			Fatty Acid & Branch Chain Amino Acid	1.6%
			Bile Acid	1.6%
			Glycerolipid	1.2%
			Fatty Acid, Acyl carnitine	1.2%
			Phospholipid	1.2%
Energy	3.9%	1	TCA Cycle	3.9%
Peptides	1.9%	1	Dipeptide	1.9%
Unknown	1.2%	1	Unknown	1.2%

Supplementary Table S2. Mass to charge ration and retention time information of internal standards.

Name of compounds	Ion mode	m/z	Retention time (min)
Creatine-(1-methyl-D3)	Positive	135.09613	0.89
Leucine-$_{13}C_6$	Positive	138.12256	2.4
L-Leucine-D10	Positive	142.16521	2.45
L-Tyrosine-13C_6	Positive	188.10183	3.24
Phenylalanine-$_{13}C_6$	Positive	288.1212	11.49
L-Tryptophan-$_{2,3,3}$-D3	Positive	208.11654	7.6
Caffeine-D_3	Positive	198.10704	7.9
N-BOC-L-Aspartic Acid	Positive	134.04478	8.6
L-Phenylalanine-13C6	Positive	172.10691	6.13
BOC-L-Tyrosine	Positive	182.08117	10.05
N-BOC-L-tert-Leucine	Positive	254.18685	10.9
N-BOC-L-Tryptophan	Positive	205.09715	11.52
Creatine-D_3	Negative	133.0805	0.95
Leucine-$_{13}C_6$	Negative	136.1069	2.41
Leucine-D_{10}	Negative	140.1496	2.6
Tyrosine-$_{13}C_6$	Negative	186.0862	3.27
Tryptophan-D_3	Negative	206.1009	7.8
N-BOC-L-Aspartic Acid	Negative	232.0827	8.6
Salicylic Acid-D_4	Negative	141.049	9.95
BOC-L-Tyrosine	Negative	280.1185	10.08
N-BOC-L-tert-Leucine	Negative	230.1393	10.9
BOC-D-Phenylalanine	Negative	166.0863	11.49
N-BOC-L-Tryptophan	Negative	303.1345	11.52

Supplementary Table S3 Parameters for MS-DIAL data processing.

Parameter name		Value	Unit
software version		4.48	
Data collection	mass accuracy (centroid parameter): MS1 tolerance	0.005	Da
	mass accuracy (centroid parameter): MS2 tolerance	0.01	Da
	retention time begin	0	min
	retention time end	100	min
	MS1 mass range begin	0	Da
	MS1 mass range end	2000	Da
	MS/MS mass range begin	0	Da
	MS/MS mass range end	2000	Da
	maximum charged number	2	
	consider CI and Br elements	FALSE	
	number of threads	4	
	execute retention time corrections	TRUE	
	together with alignment	TRUE	
Peak detection	minimum peak height	100000	amplitude
	mass slice width	0.6	Da
	minimum peak width	10	scan
	smoothing method	Linear weighted moving average	
	smoothing level	4	scan
	minimum peak width	10	scan
	exclusion mass list	None	
deconvolution	sigma window value	0.5	
	MS/MS abundance cut off	50000	amplitude
	Exclude after precursor ion	TRUE	
	Keep the isotopic ions until	0.5	Da
	keep the isotopic ions w/o MS2Dec	FALSE	
identification	msp file	combined library	
	MSP - retention time tolerance	100	min
	MSP - identification score cut off	70	%
	accurate mass tolerance (MS1)	0.005	
	accurate mass tolerance (MS2)	0.01	
	use retention time for scoring	FALSE	
	use retention time for filtering	FALSE	
	text file	SECIM library	
	TXT - retention time tolerance	0.15	min
	TXT - accurate mass tolerance	0.08	Da
	TXT - identification score cut off	85	%
	relative abundance cut off	0	%

	only report the top hit	TRUE	
	adduct - positive	[M+H]+, [M+NH4]+, [M+Na]+, [M+CH3OH+H]+, [M+K]+, [M+ACN+H]+, [M+H-H2O]+, [M+H-2H2O]+, [M+2Na-H]+, [M+IsoProp+H]+, [M+ACN+Na]+, [M+IsoProp+Na+H]+, [2M+H]+, [2M+NH4]+, [2M+Na]+, [2M+K]+, [2M+ACN+H]+, [2M+ACN+Na]+, [M+2H]2+, [M+ACN+2H]2+, [M+2ACN+2H]2+, [M+3ACN+2H]2+, [M+3H]3+, [M+P]+	
	adduct - negative	[M-H]-, [M-H2O-H]-, [M+Na-2H]-, [M+Cl]-, [M-H2O-H]-, [M+K-2H]-, [M+FA-H]-, [2M-H]-, [3M-H]-, [M-2H]2-, [M-3H]3-, [M-OH]-	
alignment	retention time tolerance	0.1	min
	MS1 tolerance	0.015	Da
	retention time factor	0.5	
	MS1 factor	0.5	
	peak count filter	20	%
	N% detected in at least one group	0	%
	remove features based on blank information	TRUE	
	sample averabe/blank average	5	fold change
	keep reference matched metabolite features	TRUE	
	keep suggested (w/o MS2) metabolite features	FALSE	
	keep removable features and assign the tag	TRUE	
	gap filing by compulsion	TRUE	
isotope tracking	tracking of isotope labels	FALSE	
normalization		LOWESS	

Supplementary Table S4 Parameters for MS-FLO data processing.

Parameter name		Value
row merging delimiter		_
contaminant ion removal: enabled		FALSE
duplicate removal	enabled	TRUE
	mz tolerance	0.003
	retention time tolerance	0.1
	peak height tolerance	3000
	minimum peak match ratio	0.9
isotope detection	enabled	TRUE
	mz tolerance	0.003
	retention time tolerance	0.1
	minimum r^2 to match	0.9
	mass shift	1.003355
adduct joiner	enabled	FALSE
	mz tolerance	0.003
	retention time tolerance	0.1
	adduct (positive)	enabled = True<br>adduct = ['M+H', 'M+NH4', 17.026547, 0.7, 0.8]<br>adduct = ['M+H', 'M+Na', 21.981942, 0.7, 0.8]<br>adduct = ['M+NH4', 'M+Na', 4.955394999999999, 0.7, 0.8]
	adduct (positive)	enabled = True<br>adduct = ['M-H', 'M+Cl', 35.976678, 0.7, 0.8]<br>adduct = ['M-H', 'M+HCOO', 46.0049, 0.7, 0.8]<br>adduct = ['M+Cl', 'M+HCOO', 24.0455, 0.7, 0.8]

Supplementary Table S5. Classification of Low abundance identified metabolites.

Class	%	Count	Contents	%
Lipids	39.6%	16	Fatty acid metabolism- acyl carnitine	3.8%
			Fatty acid, dicarboxylate	3.8%
			Fatty acid, hydroxy	3.8%
			Glycerophospholipids	3.8%
			Androgenic Steroids	1.9%
			Carnitine metabolism	1.9%
			Corticosteroids	1.9%
			Fatty acid, acy glycosides	1.9%
			Fatty acid, alpha hydroxy	1.9%
			Fatty acid, branched	1.9%
			Fatty acid, medium chain	1.9%
			Fatty acid, monohydroxy	1.9%
			Fatty acid, short chain	1.9%
			Fatty acid, straight chain	1.9%
			Glycerolipids	1.9%
			Ketone bodies	1.9%
Unknown	26.4%	11	Alkyl- phenylketones	3.8%
			Biopetrin	3.8%
			Piperidines	3.8%
			3- alkylindole	1.9%
			Benzenesulfonic acids	1.9%
			Biotin	1.9%
			Cyclic ketones	1.9%
			Flavin	1.9%
			Gamma butyrolactone	1.9%
			Hydroxybenaldehyde	1.9%
			Secondary Alcohols	1.9%
Amino Acids	15.1%	5	Creatine metabolism	3.8%
			Guanidino and acetamido metabolism	3.8%
			Tyrosine metabolism	3.8%
			delta-amino acid	1.9%
			Polyamine metabolism	1.9%
Cofactors and Vitamins	5.7%	3	Riboflavin metabolism	1.9%
			Pantothenate and CoA metabolism	1.9%
			ascorbate and aldarate metabolism	1.9%
Xenobiotics	5.7%	2	Benzoate metabolism	3.8%
			Bacterial/ fungal	1.9%
Nucleotides	3.8%	2	Pyrimidine metabolism, orotate containing	1.9%
			Ribonucleotide metabolism, imidazole containing	1.9%
Carbohydrates	1.9%	1	Glycogen metabolism	1.9%
Peptides	1.9%	1	gamma-glutamyl amino acid	1.9%

Supplementary Table S6: Metabolite-disease associations for human and bovine milk.

Class	Disease	Count	%	Total
Amino Acid	Inherited Metabolic	278	12.9	1024
	Gastrointestinal System	274	12.7	
	Cancer	92	4.3	
	Nervous System	73	3.4	
	Monogenic	68	3.2	
	Diseases < 1% Relative Abundance	57	2.6	
	Cognitive Disorder	42	1.9	
	Immune System	38	1.8	
	Urinary System	33	1.5	
	Endocrine System	30	1.4	
	Musculoskeletal System	25	1.2	
	Respiratory System	14	0.6	
Peptide	Cancer	1	0.0	3
	Gastrointestinal System	2	0.1	
Lipid	Gastrointestinal System	131	6.1	323
	Inherited Metabolic	68	3.2	
	Cancer	36	1.7	
	Immune System	23	1.1	
	Endocrine System	15	0.7	
	Diseases < 1% Relative Abundance	13	0.6	
	Nervous System	12	0.6	
	Cognitive Disorder	6	0.3	
	Musculoskeletal System	6	0.3	
	Respiratory System	6	0.3	
	Monogenic	5	0.2	
	Urinary System	2	0.1	
Cofactors and Vitamins	Gastrointestinal System	41	1.9	86
	Cancer	9	0.4	
	Diseases < 1% Relative Abundance	8	0.4	
	Immune System	7	0.3	
	Monogenic	6	0.3	
	Nervous System	6	0.3	
	Inherited Metabolic	2	0.1	
	Musculoskeletal System	2	0.1	
	Respiratory System	2	0.1	
	Urinary System	2	0.1	
	Endocrine System	1	0.0	
Xenobiotics	Gastrointestinal System	58	2.7	134
	Cancer	22	1.0	
	Inherited Metabolic	22	1.0	
	Respiratory System	11	0.5	
	Urinary System	7	0.3	
	Monogenic	4	0.2	
	Cognitive Disorder	3	0.1	
	Immune System	3	0.1	
	Diseases < 1% Relative Abundance	2	0.1	
	Endocrine System	2	0.1	

Nucleotide	Gastrointestinal System	126	5.8	375
	Inherited Metabolic	72	3.3	
	Cancer	38	1.8	
	Nervous System	36	1.7	
	Monogenic	31	1.4	
	Immune System	21	1.0	
	Diseases < 1% Relative Abundance	17	0.8	
	Urinary System	12	0.6	
	Musculoskeletal System	10	0.5	
	Cognitive Disorder	8	0.4	
	Endocrine System	2	0.1	
	Respiratory System	2	0.1	
Carbohydrate	Gastrointestinal System	48	2.2	114
	Inherited Metabolic	22	1.0	
	Cancer	14	0.6	
	Nervous System	9	0.4	
	Cognitive Disorder	6	0.3	
	Immune System	4	0.2	
	Monogenic	4	0.2	
	Urinary System	3	0.1	
	Respiratory System	2	0.1	
	Diseases < 1% Relative Abundance	1	0.0	
	Endocrine System	1	0.0	
Energy	Gastrointestinal System	24	1.1	98
	Inherited Metabolic	22	1.0	
	Nervous System	13	0.6	
	Cancer	9	0.4	
	Respiratory System	8	0.4	
	Cognitive Disorder	6	0.3	
	Monogenic	5	0.2	
	Diseases < 1% Relative Abundance	4	0.2	
	Immune System	3	0.1	
	Endocrine System	2	0.1	
	Musculoskeletal System	1	0.0	
	Urinary System	1	0.0	
	**Overall Total**			2157

Supplementary Table S7: Metabolite-disease associations for human milk.

Class	Disease	Count	%	Total
Amino Acid	Inherited Metabolic	42	11.3	155
	Gastrointestinal System	38	10.2	
	Cancer	13	3.5	
	Nervous System	11	3.0	
	Diseases < 1% Relative Abundance	9	2.4	
	Monogenic	9	2.4	
	Cognitive Disorder	8	2.2	
	Immune System	7	1.9	
	Musculoskeletal System	7	1.9	
	Endocrine System	6	1.6	
	Respiratory System	4	1.1	
	Urinary System	1	0.3	
Lipids	Gastrointestinal System	1	0.3	1
Carbohydrates	Inherited Metabolic	44	11.9	92
	Gastrointestinal System	13	3.5	
	Monogenic	12	3.2	
	Nervous System	5	1.3	
	Cognitive Disorder	4	1.1	
	Diseases < 1% Relative Abundance	3	0.8	
	Cancer	3	0.8	
	Musculoskeletal System	3	0.8	
	Endocrine System	2	0.5	
	Immune System	2	0.5	
	Urinary System	1	0.3	
Xenobiotics	Gastrointestinal System	20	5.4	48
	Inherited Metabolic	10	2.7	
	Cancer	7	1.9	
	Respiratory System	5	1.3	
	Monogenic	2	0.5	
	Urinary System	2	0.5	
	Cognitive Disorder	1	0.3	
	Immune System	1	0.3	
Nucleotide	Gastrointestinal System	19	5.1	69
	Inherited Metabolic	14	3.8	
	Cancer	7	1.9	
	Monogenic	7	1.9	
	Musculoskeletal System	5	1.3	
	Nervous System	5	1.3	
	Immune System	4	1.1	
	Urinary System	3	0.8	
	Respiratory System	2	0.5	
	Diseases < 1% Relative Abundance	1	0.3	
	Cognitive Disorder	1	0.3	
	Endocrine System	1	0.3	
Energy	Respiratory System	2	0.5	6
	Diseases < 1% Relative Abundance	1	0.3	

	Cancer	1	0.3	
	Gastrointestinal System	1	0.3	
	Nervous System	1	0.3	
	**Overall Total**			371

Supplementary Table S8: Metabolite-disease associations for bovine milk.

Class	Disease	Count	%	Total
Nucleotide	Gastrointestinal System	20	19.6	58
	Cancer	13	12.7	
	Reproductive System	7	6.9	
	Nervous System	5	4.9	
	Diseases < 1% Relative Abundance	3	2.9	
	Monogenic	3	2.9	
	Urinary System	3	2.9	
	Immune System	2	2.0	
	Inherited Metabolic Disorder	2	2.0	
Lipids	Gastrointestinal System	17	16.7	36
	Inherited Metabolic Disorder	10	9.8	
	Immune System	4	3.9	
	Cancer	2	2.0	
	Endocrine System	2	2.0	
	Nervous System	1	1.0	
Amino Acids	Inherited Metabolic Disorder	4	3.9	8
	Gastrointestinal System	3	2.9	
	Cancer	1	1.0	
	**Overall Total**			102
